# Drug coated balloons for coronary artery bifurcation lesions: A systematic review and focused meta-analysis

**DOI:** 10.1371/journal.pone.0251986

**Published:** 2021-07-09

**Authors:** Natasha H. Corballis, Sophie Paddock, Tharusha Gunawardena, Ioannis Merinopoulos, Vassilios S. Vassiliou, Simon C. Eccleshall

**Affiliations:** 1 Department of Cardiology, Norfolk and Norwich University Hospital NHS Foundation Trust, Norwich, United Kingdom; 2 Norwich Medical School, Bob Champion Research and Education, University of East Anglia, Norwich, United Kingdom; University of Tampere, FINLAND

## Abstract

**Objectives:**

We sought to systematically review the evidence supporting the role of drug coated balloons (DCBs) in the treatment of coronary bifurcation lesions.

**Background:**

DCBs are emerging as an attractive alternative treatment strategy for treating coronary bifurcations due to simplifying the approach and reducing rates of stent related complications. We systematically reviewed the evidence for DCB use in coronary bifurcations and conducted a focused meta-analysis on late lumen loss in the side branch comparing DCB and plain old balloon angioplasty (POBA).

**Methods:**

This study was conducted in line with the PRISMA statement. All studies (including both RCTs and observational studies, excluding case reports) using DCB as part of a bifurcation strategy were included in this review. A literature search identified a total of ten studies for inclusion. A focused meta-analysis was undertaken for the use of DCB in side-branch compared with POBA. Mean late lumen loss was used with a random effects model due to heterogeneity.

**Results:**

DCB was found to be superior to POBA for side branch treatment in bifurcations (p = 0.01). There are four studies that investigated the use of DCB for main branch treatment in a bifurcation, with evidence supporting its safety in main branches of bifurcation lesions, while prospective observational studies have demonstrated favourable target lesion revascularisation rates.

**Conclusion:**

Although there is a lack of robust RCTs comparing DCBs with current generation DES, DCBs appear safe in main branch bifurcation lesions with improved side branch late lumen loss when compared with DES or POBA.

## Introduction

A coronary bifurcation lesion is defined as a lesion occurring at, or adjacent to, a significant division of a major epicardial vessel [1]. Bifurcation lesions account for 1 in 5 of all cases requiring percutaneous coronary intervention (PCI) [1] and are associated with worse outcomes than non-bifurcation PCI [2]. Treatment strategies for these lesions are complex and there remains a lack of consensus on the best approach. We therefore systematically reviewed the evidence supporting the use of drug coated balloons (DCBs) as an alternative to complex stenting.

The current European Society of Cardiology (ESC) guidelines [3] recommend main branch-only stenting with provisional side-branch stenting as the preferred strategy due to reduced procedure time, contrast load, radiation dose and a lack of evidence supporting superiority of a two-stent strategy [3, 4]. The European Bifurcation Club also supports the use of a main branch-only stenting in the majority of cases with provisional side branch stenting only if required due to severe side-branch recoil or flow limitations after stenting the main branch [1].

Given the complexity of coronary bifurcation anatomy with significant size mismatch between proximal and distal main branch which may drive rates of instent restenosis [5] and the potential impact of main branch stenting affecting side-branch coronary flow dynamics [6], the concept of leaving no permanent implant behind is appealing.

DCBs are a standard semi-compliant angioplasty balloon, coated in a cytotoxic chemotherapeutic agent, most commonly paclitaxel. Their use in angioplasty is currently recommended by ESC guidelines for in-stent restenosis only [3]. There is increasing evidence supporting their use in de novo small and large vessels [7–9]. Their use in bifurcation lesions is appealing as it would provide a more straight-forward treatment strategy, theoretically reduce rates of lesion thrombosis [1] which drive adverse outcomes, side-branch re-stenosis which occur in up to 10% [10] and prevent loss of side branches due to the lack of stent strut jailing. With the guidelines and evidence supporting the KISSS (keep it swift, simple and safe) principle [11], DCBs appear an attractive alternative. The European Bifurcation Club’s latest meeting has highlighted DCBs as an area of interest for bifurcation PCI [12] with a recent international DCB consensus group highlighting the role of DCBs in bifurcation lesions [13].

As such, we sought to systematically review the existing literature for the use of DCBs in coronary bifurcation lesions.

## Materials and methods

The study was conducted according to the Preferred Reporting Items for Systematic Reviews and Meta-Analyses statement (PRISMA). A systematic search was conducted on PubMed (1946- 10^th^ August 2020) and Scopus by two independent researchers, using the MeSH search terms “drug coated balloon”, “drug eluting balloon”, “PCI” and “bifurcations” in August 2020 and a further review of the references of the relevant papers were included. All study designs that used a drug coated balloon in treatment of a coronary bifurcation lesion (either main branch, side branch or combined strategy) were included. This included randomised controlled trials, prospective observational studies and both prospective and retrospective registries. Case reports were excluded. Only full studies were included. All clinical presentations (stable angina, acute coronary syndromes including STEMI) were included. The exclusion criteria were any study not assessing the use of drug coated balloon for treating a coronary bifurcation lesion. The primary outcome measures included MACE and angiographic follow-up measures encompassing late lumen loss and binary restenosis rate using quantitative coronary angiography. The review was not registered with PROSPERO.

Two independent researchers (NC and SP) extracted data from downloaded PDFs of studies into a pre-tabulated excel spreadsheet. This included data on 1) publication details (notably, study reference, main author, year of publication), 2) study design and methodology, 3) participants including baseline characteristics and sample size and 4) outcomes. Baseline patient characteristics that were extracted included: mean age, sex, risk factors including smoking, hypertension, diabetes, dyslipidaemia, MI, previous PCI and clinical presentation. The lesion characteristics extracted included vessel treated and Medina Classification. Clinical outcomes recorded included major adverse cardiovascular outcomes (MACE), target lesion revascularisation and angiographic outcomes including late lumen loss, angiographic restenosis and mean lumen area. Due to heterogeneity of study design, a systematic review was undertaken, but a subgroup meta-analysis has also been conducted for DCB use in the side branch (using a random effects model) but the primary aim was to systematically review the literature on the use of DCBs in coronary bifurcations.

The Cochrane Risk of Bias Assessment tool (RoB 2) was used to identify quality of included papers for the randomised controlled trials, covering five domains: 1. The randomisation process, 2. Deviations from the intended interventions, 3. Missing outcome data 4. Measurement of the outcome and 5. Selection of the reported result. This was conducted by two independent investigators. Statistical analysis was conducted using Review Manager 5.3 for MacOS with summary statistics from each study expressed as mean lumen loss, before combining these statistics from each study, using a random effects model with difference between the two outcomes expressed as mean difference with 95% confidence intervals, and I^2^ to assess for heterogeneity. Statistical significance was set at p<0.05.

## Results

**Fig 1:**
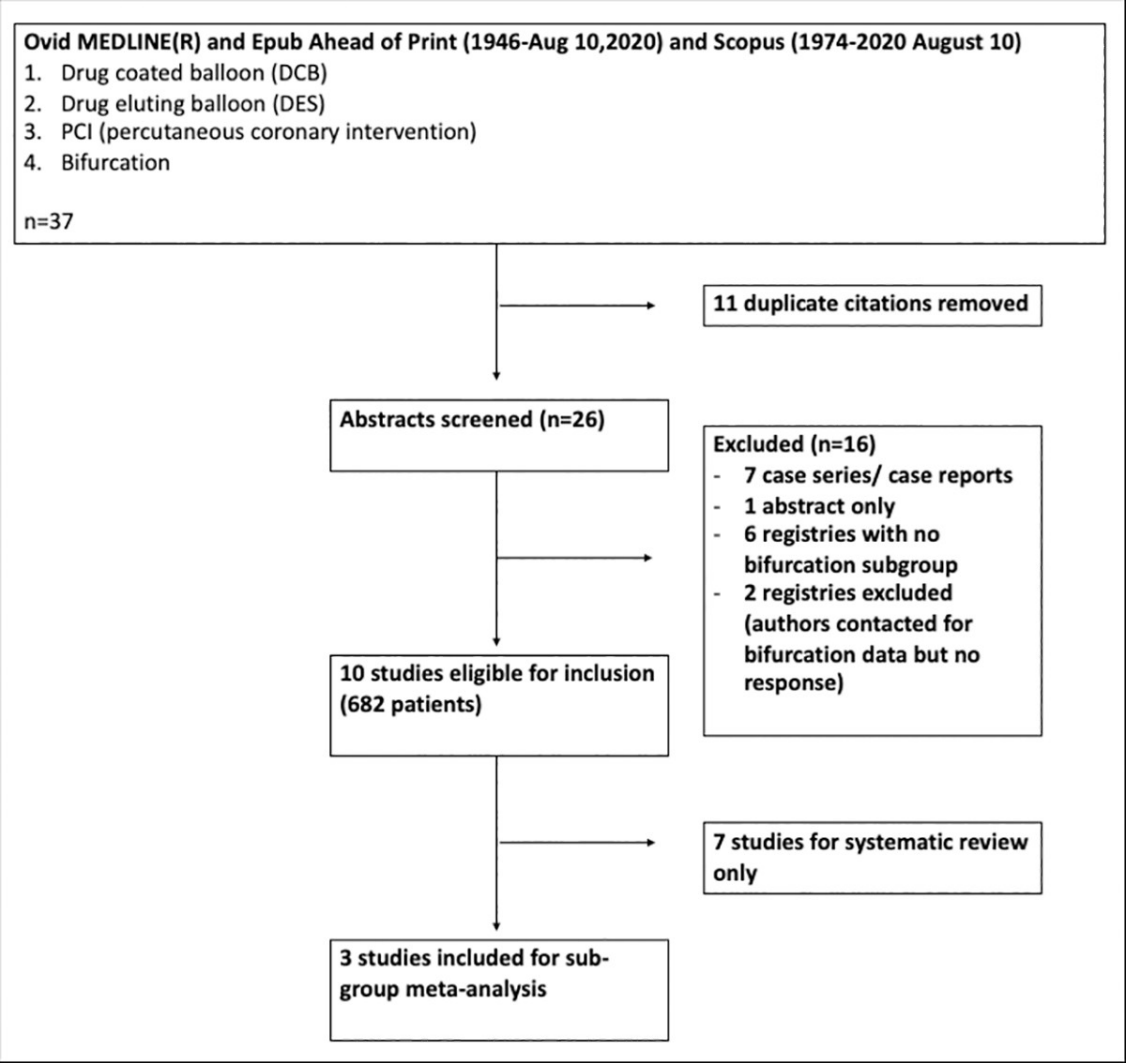
Search strategy.

Of 37 papers identified in the initial search, 12 papers were included in analysis. Two registry trials were for all-comer DCB use, and the investigators were contacted for outcomes for subgroup analysis for bifurcation lesions although such data was unavailable. Fig 1. represents the search strategy, in accordance with PRISMA guidelines.

### Patient characteristics

As shown in Table 1, the majority of patients were male (75.4%) with a mean age of 64.9 (±10.7). The most frequently occurring risk factors were hypertension (70.5%) and dyslipidaemia (63.8%). The majority of studies featured predominantly patients with stable angina/ unstable angina, with several including acute MI in their exclusion criteria.

**Table 1:** Patient characteristics for all included studies.

**First author/ trial/ reference**	**Year**	**Study design**	**Intervention**		**Sample size**	**Age**	**Male**	**Smoking**
PEPCAD-V [14]	2011	Prospective observational study	BMS to MB and DCB to SB		28	65.8±9.6	20 (71.4%)	15 (53.6%)
DEBIUT [15]	2012	RCT	BMS MB/DCB SB		117	63.6±7.8	85 (72.6%)	69 (58.9%)
			BMS MB/ POBA SB					
			DES MB/POBA SB					
Herrador et al. [16]	2013	RCT	DCB v POBA to SB		100	62.5±10.6	83 (83%)	52 (52%)
BABILON [17]	2014	RCT	MB BMS and SB DCB v. MB DES and SB POBA		108	64.8±11.2	70 (64.8%)	54 (50%)
Schulz et al [18]	2014	Prospective observational study	DCB only		38	70.7±11.9	23 (60.5%)	ND
BIOLUX-I [19]	2015	Prospective observational study	DES to MB and DCB to SB		35	65.9 ±9.5	29 (82.8%)	26 (74.3%)
PEPCAD-BIF [20]	2016	RCT	DCB v POBA		64	67±11	47 (73.4%)	16 (25%)
Bruch et al [21]	2016	Prospective registry	DCB only (bailout stenting if required)		127	66.1±10.1	102 (80%)	43 (33.9%)
Vaquerizo et al [22]	2016	Prospective registry	DCB only to SB		49	61.8±11.8	38 (77.6%)	22 (44.9%)
Her et al [23]	2016	Prospective observational study	DCB to MB with OCT to assess SB		16	60.3±6	11 (68.8%)	6 (37.5%)
**First author/ trial/ reference**	**DM**	**HTN**	**Dyslipidaemia**	**Family history**	**Previous MI**	**Previous PCI**	**Stable angina**	**ACS**
PEPCAD-V	4 (14.3%)	24 (85.7%)	17 (60.7%)	10 (35.7%)	5 (17.9%)	ND	28 (100%)	0 (0%)
DEBIUT	13 (11.1%)	67 (57.5%)	65 (55.6%)	ND	25 (21.4%)	38 (32.5%)	ND	ND
Herrador et al	33 (33%)	62 (62%)	56 (56%)	ND	12 (12%)	9 (9%)	24 (24%)	76 (76%)
BABILON	34 (31.5%)	67 (62%)	69 (63.9%)	ND	27 (25%)	19 (17.6%)	ND	47 (43.5%)
Schulz et al	17 (44.7%)	35 (92.1%)	20 (52.6%)	ND	ND	ND	21 (53.8%)	18 (46.2%)
BIOLUX-I	8 (22.9%%)	24 (68.6%)	29 (82.8%)	14 (41.2%)	ND	8 (22.9%)	29 (82.8%)	
PEPCAD-BIF	23 (35.9%)	ND	ND	ND	12 (18.8%)	ND	41 (64.1%)	15 (23.4%)
Bruch et al	40 (31.5%)	116 (91.3%)	96 (75.6%)	ND	ND	ND	69 (54.3%)	21 (16.5%)
Vaquerizo et al	20 (40.8%)	26 (53.1%)	30 (61.2%)	ND	8 (16.3%)	16 (32.7%)	ND	30 (65.3%)
Her et al	4 (25%)	7 (43.8%)	8 (50.0%)	2 (12.5%)	ND	ND	11 (68.6%)	5 (31.2%)

Where age is expressed as mean±standard deviation, and all other numbers are presented as number (%). MB = main branch, BMS = bare metal stent, DCB = drug coated balloon, SB = side-branch, DES = drug eluting stent, POBA = plain old balloon angioplasty, RCT = randomised controlled trial, DM = diabetes mellitus, HTN = hypertension, ND = not disclosed.

### Angiographic/ Lesion characteristics

Of a total of 688 lesions included, the majority of lesions treated were left anterior descending (LAD)/ diagonal (DG) bifurcations (63.4%), with 25.3% accounting for circumflex (Cx)/ obtuse marginal (OM) bifurcations and 8% right coronary artery (RCA)/ posterior descending artery (PDA) bifurcations. Some seven of the included studies excluded left main stem (LMS) disease so this only accounted for 3.3% of lesions treated. The Medina Classification is summarised in Table 2.

**Table 2:** Lesion characteristics for included studies.

First author/ trial/ reference	Bifurcation treated				Medina Classification						
	LMCA	LAD	Cx	RCA	1,1,1	1,1,0	1,0,1	0,1,1	1,0,0	0,1,0	0,0,1
PEPCAD-V		19 (67.8%)	9 (32.2%)		9 (32.1%)	1 (3.6%)	3 (10.7%)	8 (28.6%)	2 (7.1%)	3 (10.7%)	1 (3.6%)
DEBUIT	N/A	98 (83.8%)	15 (12.8%)	3 (2.6%)	ND	ND	ND	ND	ND	ND	ND
Herrador et al.	10 (10%)	54 (54%)	25 (25%)	10 (14%)	ND	ND	ND	ND	ND	ND	ND
BABILON	N/A	69 (63.9%)	28 (25.9%)	11 (10.2%)	62 (57.4%)	ND	ND	ND	ND	ND	ND
Schulz et al.	13 (33.3%)	11 (28.2%)	8 (20.5%)	7 (17.9%)	7 (17.9%)	4 (10.3%)	1 (2.6%)	7 (17.9%)	2 (5.1%)	6 (15.4%)	12 (30.8%)
BIOLUX-I	N/A	29 (82.8%)	3 (8.6%)	3 (8.6%)	7 (20%)	14 (40%)	1 (2.8%)	3 (8.6%)	5 (14.3%)	5 (14.3%)	0 (0%)
PEPCAD-BIF	N/A	14 (43.8%)	16 (50%)	2 (6.3%)	N/A	N/A	N/A	2 (6.3%)	N/A	3 (9.4%)	27 (84.4%)
		2 (6.3%)	3 (9.4%)	5 (15.6%)				24 (75%)		17 (53.1%)	13 (40.6%)
Bruch et al.	N/A	78 (60%)	43 (33.1%)	9 (6.9%)	60 (46.2%)	16 (12.3%)	15 (11.5%)	21 (16.3%)	4 (3.1%)	5 (3.8%)	9 (6.9%)
Vaquerizo et al	N/A	26 (50%)	13 (24.6%)	4 (8.2%)	N/A	N/A	N/A	N/A	N/A	N/A	49 (100%)
Her et al.	N/A	21 (80.8%)	1 (3.8%)	4 (15.4%)	7 (26.9%)	6 (23.1%)	2 (7.7%)	N/A	4 (15.4%)	7 (26.9%)	N/A

In Table 2, LMCA = left main coronary artery, LAD = left anterior descending artery, Cx = circumflex artery, RCA = right coronary artery.

### DCB for side-branch only

PEPCAD-V (2011) [14] paved the way for the use of DCB in side-branch of bifurcation lesions as a first-in-man observational study with the use of bare metal stent (BMS) in the main branch, reporting a mean late lumen loss at 9 month angiography of 0.21±0.48.

DEBIUT (2012) [15], Herrador et al. [16] and BABILON (2014) [17] all compared the performance of DCB to the side-branch of a bifurcation lesion with either plain old balloon angioplasty (POBA) or DES, all using late lumen loss (LLL) at angiographic follow-up as primary outcomes.

**Fig 2:**
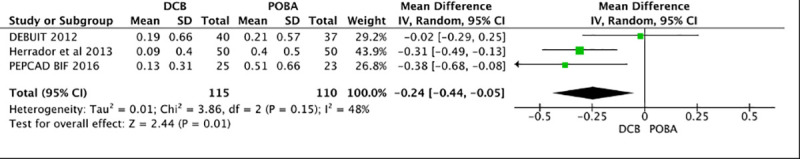
Forest plot for late lumen loss in DCB v POBA for side branch treatment.

We conducted a subgroup meta-analysis for side branch late lumen loss in these three studies (n = 281), as seen in Fig 2. This showed a statistically significant difference favouring DCB over POBA (p = 0.01). A random effects model was used. Of interest, Herrador et al. was the only trial individually to show statistically significant benefit of DCB over POBA and this was the only study design that used a DES in the main branch rather than BMS.

Of the non-randomised, prospective single-arm trials, BIOLUX-1 (2015) [19] and Vaquerizo et al. [22] both reported favourable late lumen loss at follow up angiography for DCB to side-branch. For BIOLUX-1 (n = 28), mean LLL for side-branch was 0.1±0.43 [19] and Vaquerizo (n = 31), LLL for SB was 0.32±0.73, although notably, bailout stenting in this cohort was high at 14% [22].

### DCB as a main branch (MB) strategy

Schulz et al. [18] performed a single arm, prospective observational study looking at a DCB only strategy for bifurcations (n = 39). Of these, 33.3% were LMS bifurcation lesions and 46% were true bifurcation lesions (1,1,1; 1,1,0 or 0,1,1). With a primary outcome of angiographic restenosis at follow-up angiography, 3 (10% as n = 30) had angiographic evidence of restenosis- all of these were LMS disease [18].

PEPCAD-BIF (2016) randomised patients (n = 64) to either DCB or POBA only approach for bifurcation lesions that did not incorporate proximal main branch disease (i.e. Medina 0,1,0, 0,0,1 or 0,1,1). It incorporated largely small vessels (mean vessel diameter 2.4mm). This showed a statistically significant improvement in late lumen loss favouring the DCB arm (0.13 v 0.51, p = 0.013) [20]. As the first DCB only randomised trial, this showed promising results for the use of DCB only bifurcation.

Bruch et al. [21] conducted a prospective registry of a DCB only strategy for coronary bifurcation lesions of any medina classification. Of these, 97 (74.6%) were a true bifurcation lesion and the mean vessel diameter of the main branch was 2.98. The primary outcome was target lesion revascularisation (TLR) at 9 months with a TLR rate of 3 (4.5%) with a cumulative MACE of 4 (6.1%). These outcomes show the promise of a DCB only strategy although there was a high bailout stenting rate of 45%.

Her et al. [23] performed a single centre, prospective observational study (n = 16) using a DCB only approach for the main branch with a primary outcome of OCT lumen area of both main branch and side branch at angiographic follow-up. There was a significant increase in side-branch mean lumen area at 9 months (0.92–1.42, p = 0.013) with similar increase in main branch mean lumen area (4.77–5.69, p = 0.008) [23]. Table 3 summarises the results from all studies not included in the focused meta-analysis.

**Table 3:** Summaries of results from all studies not included in the focused meta-analysis.

First author/ trial/ reference	Study design	Primary outcome	Result
PEPCAD-V [14]	Prospective observational study	LLL at 9 months	MV LLL: 0.38 ± 0.46 mm
			SB LLL: 0.21 ± 0.48mm
Schulz et al [18]	Prospective observational study	Binary restenosis at 4 months	Re-stenosis: 10%
BIOLUX-I [24]	Prospective observational study	Side-branch LLL at 9 months	SB LLL: 0.1 ± 0.43 mm
PEPCAD-BIF [20]	RCT	LLL at 9 months	LLL: 0.13 v 0.51 mm (p = 0.001)
Bruch et al [25]	Prospective observational study	TLR at 9 months	TLR for DCB only: 4.5%
Vaquerizo et al [22]	Prospective registry	MACE at 12 months	MACE: 16.3%
Her et al [23]	Prospective observational study	Side-branch ostial lumen area at 9 months (OCT)	Mean gain: 0.6 ± 0.93 mm^2^

Where LLL = late lumen loss, MV = main vessel, SB = side branch, RCT = randomised controlled trial, TLR = target lesion revascularisation, MACE = major adverse cardiovascular outcomes, OCT = optical coherence tomography.

## Discussion

The cardiology community still remains uncertain as to the best strategy to treat coronary bifurcations. The subgroup meta-analysis shows that DCB is superior to POBA for side branch only treatment in bifurcation disease with regards to late lumen loss at follow-up angiography. Individually, only one study showed statistical significance which may be due to the use of stents across the ostium of the main branch in all cases and particularly, the use of BMS as opposed to DES in the DCB arms which is likely to drive any increase in MACE rates. As there is now evidence supporting the use of DCBs in small vessel coronary disease [8], it follows that DCBs would be an effective treatment option for side-branch lesions in coronary bifurcations, and this has been confirmed in this sub-group meta-analysis.

The evidence supporting a DCB only bifurcation strategy is of interest. The results of PEPCAD-BIF [20] showed DCB is superior to POBA for main branch bifurcation lesions, although this only looked at DCB use in either the distal main branch or side-branch, but it did include both small and large vessels. Bruch et al [25] also add strength to the argument for a DCB only strategy, with promising MACE rates of 6.1%, whereas a comparable lesion complexity DES cohort reports a MACE of 20.8% for a simple stent strategy [11]. The results of Her et al [23] are of particular interest. Although this is a small patient group, and a single arm observational study, it confirms that when a DCB is used to treat the main branch, there is a significant increase in the ostial side-branch area at follow-up OCT. This is of benefit in reducing the high rates of ISR currently associated with side-branches in bifurcation lesions [26]. With regards to cost-effectiveness, although the shelf-cost of a DCB is higher than a DES, the reduced procedural complexity requiring less equipment, particularly with a two stent strategy including kissing balloon inflation makes DCB a cost-effective alternative, although no formal cost-effectiveness analysis has been undertaken. In addition, in our practice we find that with DCB less intravascular imaging is required making it overall cheaper for this type of patients.

Based on this review, and our experience with the use of DCBs in bifurcation lesions, we would suggest the following strategies to treating a bifurcation lesion with a DCB strategy. If the operator’s preference is a DES approach to the main vessel, the use of a DCB for the side branch can be used upfront (prior to stenting) if there is significant disease of the side branch. The benefit of this over POBA has been confirmed by our sub-group meta-analysis. If the side branch is not significantly diseased and the treatment strategy is to treat the main vessel only, a DCB can be used to the side branch after stenting if the flow becomes compromised as a result of stenting.

If the operator’s preference is a DCB only strategy, either due to anatomical or patient factors, a provisional main vessel approach with a DCB is a good initial option. On the registry evidence presented above, a DCB only strategy appears safe in the main vessel. From Her et al’s OCT study [23], if there is pinching of the ostium of the side branch after DCB to the main vessel, there is no necessity to pursue this as the side branch ostium lumen will increase due to positive remodelling. If there is an indication to treat the side branch too (e.g. long segment of side branch disease, large vessel, significant territory supplied) [3], then a sequential DCB to side branch followed by DCB to main vessel would be a reasonable approach. Based on current DCB consensus guidelines [13], there is usually no need for kissing balloon inflation. The only time when this could be indicated is if there is loss of flow down either vessel after ballooning in order to restore carinal bifurcation geometry and integrity.

## Limitations

Bifurcation studies are difficult to interpret given the lesion variability which will influence outcomes and is neither easily describable or accountable for. These factors include the bifurcation angle, the significance of the side-branch, the extent of main branch disease and the main branch/ side branch size mismatch, none of which is encompassed in Medina classification. Despite a wealth of RCTs for DES strategies in bifurcations, uncertainties still remain in the best stenting strategy. In comparison, there are a small number of studies looking at DCBs in bifurcation lesions. The use of BMS in conjunction with DCB in some of the earlier RCTs also will influence interpretation of results. As BMS are associated with significantly higher rates of TLR, stent thrombosis (ST) and MI when compared with DES [27], in order to understand the performance of DCBs in bifurcations, it needs to be compared with current generation DES.

## Conclusion

In summary, it appears that DCBs could be a potential alternative treatment strategy for bifurcations (both main branch and side-branch) with demonstrated safety. However, there remains a paucity of large registry data confirming their efficacy or RCTs comparing their use to a DES strategy.

## Supporting information

S1 Checklist (DOCX)
